# A Review Into the Effects of Pamidronic Acid and Zoledronic Acid on the Oral Mucosa in Medication-Related Osteonecrosis of the Jaw

**DOI:** 10.3389/froh.2021.822411

**Published:** 2022-02-09

**Authors:** George Bullock, Cheryl A. Miller, Alasdair McKechnie, Vanessa Hearnden

**Affiliations:** ^1^Department of Materials Science and Engineering, Kroto Research Institute, The University of Sheffield, Sheffield, United Kingdom; ^2^School of Clinical Dentistry, The University of Sheffield, Sheffield, United Kingdom; ^3^School of Dentistry, University of Leeds, Leeds, United Kingdom

**Keywords:** bisphosphonate-related osteonecrosis of jaw, pamidronate, zoledronate, oral mucosa, wound healing

## Abstract

Medication-related osteonecrosis of the jaw (MRONJ) is a growing problem without an effective treatment, presenting as necrotic bone sections exposed via lesions in the overlying soft tissue. There is currently a lack of clarity on how the factors involved in MRONJ development and progression contribute to disease prognosis and outcomes. Bisphosphonates (BPs), the most common cause of MRONJ, affect bone remodeling, angiogenesis, infection, inflammation and soft tissue toxicity, all of which contribute to MRONJ development. This article reviews the cellular mechanisms through which BPs contribute to MRONJ pathology, with a focus on the effects on cells of the oral mucosa. BPs have been shown to reduce cell viability, reduce proliferation, and increase apoptosis in oral keratinocytes and fibroblasts. BPs have also been demonstrated to reduce epithelial thickness and prevent epithelial formation in three-dimensional tissue engineered models of the oral mucosa. This combination of factors demonstrates how BPs lead to the reduced wound healing seen in MRONJ and begins to uncover the mechanisms through which these effects occur. The evidence presented here supports identification of targets which can be used to develop novel treatment strategies to promote soft tissue wound healing and restore mucosal coverage of exposed bone in MRONJ.

## Introduction

Medication-related osteonecrosis of the jaw (MRONJ) is a disease that presents as necrotic bone exposed via lesions in the soft tissues, and is currently without an effective treatment [1]. It is encountered in patients receiving anti-resorptive or anti-angiogenic medications, most often in patients receiving bisphosphonates (BPs), used to treat osteoporosis, bony metastases and hypercalcaemia of malignancy. Increasingly, bisphosphonates are also used to prevent bone metastases [2]. BPs are the most commonly prescribed anti-resorptives worldwide [3] with over 190 million prescriptions for oral BPs estimated to have been dispensed worldwide since their first clinical use in 1969 [4]. In 2015, it was estimated that per year, 10 new patients per million population suffer with MRONJ, drastically reducing their quality of life; affecting their ability to speak, eat and function normally in society [5]. The prevalence of MRONJ increases every year alongside BP prescription numbers [6], and millions of individuals are at risk. Approximately 15% of patients treated for metastases with BPs are estimated to develop MRONJ [7] and there is an urgent unmet clinical need to improve outcomes for MRONJ patients.

The current diagnostic criteria for MRONJ were defined in 2014 by the American Association of Oral and Maxillofacial Surgeons (AAOMS) [8]. A patient is defined as having MRONJ when they have “exposed bone or bone that can be probed through a fistula(e) that has persisted for more than eight weeks,” provided they are being or have been treated with anti-resorptive, anti-angiogenic or other medications including a number of monoclonal antibody therapies [9], and have no history of radiation therapy to the jaws [1]. Within this definition of MRONJ, there are four stages of increasing severity (Stages 0–3), beginning at Stage 0 where no clinical evidence of necrotic bone is present, up to Stage 3 where severe complications exist (e.g., fractures, extra oral fistulae, necrosis extending beyond the alveolar region). While these standardized criteria are useful to define severity, more recently it has been found that the AAOMS diagnostic criteria may leave up to 25% of MRONJ patients undiagnosed [10]. It has been recommended that the diagnostic criteria are updated to be more comprehensive, to enable diagnosis at an earlier stage in the disease progression and to support earlier intervention [11].

Due to the relative recency of the classification of MRONJ, its pathophysiology and the risk factors associated are not fully clear, and studies have been focussed on elucidating these areas [11–15]. Once triggered, effects on bone remodeling, angiogenesis, inflammation, infection, and toxicity to the soft tissue are thought to combine in the progression of the disease through the stages of the AAOMS diagnostic criteria [1]. Soft tissue toxicity has been hypothesized to be key to this progression [16], with the exposure of the necrotic bone through lesions in the soft tissue increasing infection risk [17].

The relatively low incidence and incomplete knowledge of MRONJ pathogenesis has made devising a definitive treatment challenging [18] and strategies vary depending on the patients other co-morbidities, quality of life, and stage of the disease, and include pain medication, antibiotics and antibacterial rinses, and in more severe cases, the surgical debridement of necrotic tissue [19–21]. A drug holiday has been advised previously, however, in a retrospective study of risk factors, this was shown to be ineffective in the prevention of MRONJ [22]. However, recent literature has indicated a reduced MRONJ-risk, including improved soft tissue healing, from a drug holiday immediately prior to tooth extraction in a minipig model of MRONJ [23], which warrants further investigation.

Due to the high clinical need, there has been a large amount of research in the field since the discovery of MRONJ. Particular focus has been given to further understanding the pathophysiology of the disease, in particular with regards to BPs, as the most prevalent cause of MRONJ, and their effect on soft tissues, given its importance in disease progression [16].

### Aim

The aim of this study was to scientifically review the literature investigating the mechanism of action of BPs on the oral mucosa *in vitro*. This review aims to examine how BPs affect the ability of oral keratinocytes and fibroblasts to restore mucosal coverage and achieve wound healing, thereby examining the role of BPs and soft tissue toxicity in MRONJ development.

## Bisphosphonates

### Mechanism of Action

MRONJ has been caused by a wide range of medications, with anti-resorptives and anti-angiogenic medications, most common. The most prominent of these medications are BPs, a group of anti-resorptive drugs, with the US market for BPs reported at $11–12 billion in 2012 [24]. Other medications linked to MRONJ development are denosumab [25], an anti-resorptive which prevents bone resorption by inhibiting Receptor activator of nuclear factor kappa-β ligand (RANKL), and with a risk of MRONJ comparable to ZA [1], and anti-angiogenic medications prescribed as cancer treatments, such as sunitinib (a tyrosine kinase inhibitor) and bevacizumab (a vascular endothelial growth factor (VEGF) inhibitor) [26]. These medications are often prescribed alongside BPs, and can raise the risk of MRONJ by as much as 10% [1]. Due to the prevalence of BP-related MRONJ and the research in the area mainly focussing on BPs, this review will focus on BPs alone.

**Figure 1:**
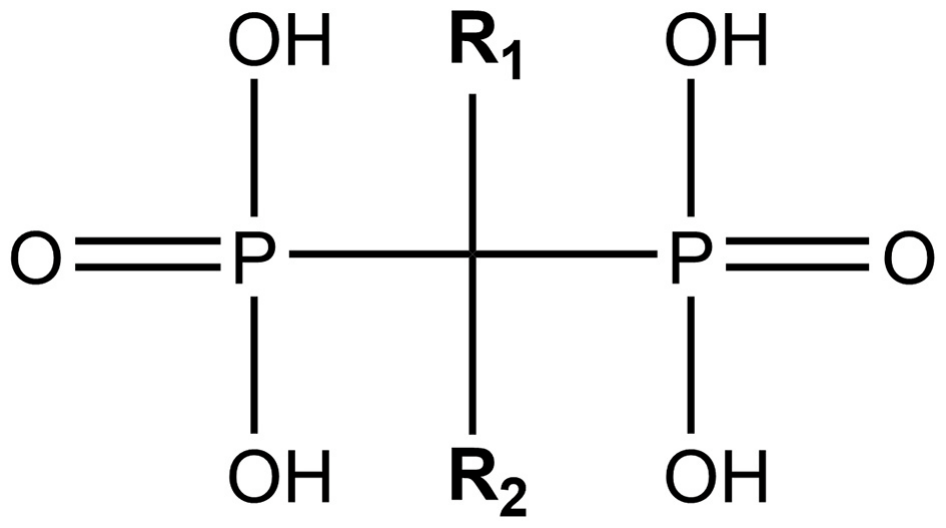
The generic structure of a bisphosphonate. This figure was created by using ChemDraw software.

All BPs have the same generic structure, shown in Figure 1, with two phosphonate molecules bound to a non-hydrolysable carbon [27], and this structure gives the BPs their specific affinity for bone [17, 28]. BPs also have two functional groups, which affect the mechanism of action and anti-resorptive potency of the drugs [17, 27, 29]. Modern BPs (e.g., pamidronic acid (PA) and ZA, respectively), contain nitrogen in the R_2_ functional group, and are therefore also known as nitrogen-containing BPs (nBPs) [17]. These block the production of farnesyl pyrophosphate synthase in the mevalonate pathway [30], a pathway important for protein prenylation. Inhibition of this pathway prevents proteins from acting at the correct location in the cell. This leads to changes in many cellular processes including cytoskeletal organization, vesicular trafficking and apoptosis [28, 31–34].

Alongside the primary effect on bone resorption, BPs also effect other cells and tissues. These secondary effects allow for BPs to decrease tumor burden in patients with metastases, with hypothesized direct and indirect effects including reduced cell invasion, adhesion and angiogenesis, and increased apoptosis [35–39]. These secondary effects, however, also contribute to MRONJ development.

The full pathophysiology of MRONJ is yet to be determined [20], however, several factors are thought to combine in disease development, affecting both the hard and soft tissues. BPs affect bone remodeling, angiogenesis, infection, inflammation and the soft tissue, and all these effects are thought to contribute to disease development after the initial trigger [1]. The soft tissue has previously been hypothesized as a key part of the development process [16] and, as such, a large amount of research has been conducted in this area.

Infection is now thought key to MRONJ development [11], and bacteria are commonly found in biopsied necrotic tissue [40, 41]. As MRONJ occurs in the mouth, and as the wound becomes exposed, the risk of bacterial infection is increased [13, 42] while inflammation has been linked to MRONJ development, with it hypothesized that early, low level inflammation may be a trigger for the condition [43]. Infection and chronic inflammation are known to limit the ability of epithelial tissues to heal efficiently and effectively while oral mucosa wound healing is known to be more difficult without healthy underlying bone [44]. The combination of the BP effects on these biological processes all contribute to the mucosal damage and limited wound healing observed in the oral mucosa of MRONJ patients.

### Effects on the Oral Mucosa

In current surgical treatment, closing the mucosa to restore the soft tissue barrier and reduce residual bone exposure is thought to be critical for disease resolution [11]. Around 75% of MRONJ cases affect the mandible [1], which has a thinner mucosa compared to the maxilla and may therefore be more susceptible to damage [45]. As the bone is damaged and exposed to the surrounding environment (through dental surgery, trauma or infection), BPs can be released which at sufficient concentrations are toxic to the surrounding tissues [16]. The full mechanism by which BPs are released into the mouth, and the concentrations the oral mucosa is exposed to are not known, and is difficult to estimate given the variety of BPs, affinities and dosing regimen [46]. A study by Scheper et al. measured the ZA concentration in the saliva of BP patients 5 min after IV treatment at between 0.4 and 5 μM [47], whilst, the bone concentration is thought to be between 200 μM and 2 mM [46]. In higher affinity BPs, such as ZA, more BP will be present and it will be located close to the surface of the bone [29] while the concentration of BPs with a lower calcium affinity is expected to be lower. As with infection, wound healing can reduce the pH of the mouth and therefore this could potentially release more BPs into the local environment [48].

Due to the severity of MRONJ and the increasing number of patients diagnosed every year [6], there is an increasing amount of research aiming to further understand the disease. A key focus is the effects of BPs on the oral mucosa, and research in the area includes both 2D and 3D cell culture, and a wide variety of BP effects, including viability, migration, proliferation and adhesion have been studied. This section only reviews articles which have used either pamidronic acid (PA) or zoledronic acid (ZA), as these BPs have been commonly associated with MRONJ development and have the largest body of literature [49, 50]. PA has an intermediate anti-resorptive potency, while ZA is one of the highest potency BPs. Other BPs have been examined within some of the literature reviewed, and the anti-resorptive potency of the BP generally correlates with its toxicity to the oral mucosa [51–53]. This section therefore presents a demonstration of the effects of BPs on the soft tissue which can be extrapolated for other BPs.

Studies mainly focus on two cell types key to oral mucosa wound healing: fibroblasts and keratinocytes. Fibroblasts exist in the lamina propria and synthesize the extracellular matrix (ECM) while keratinocytes make up the stratified squamous epithelium. In wound healing, keratinocytes detach from the wound edges to migrate and proliferate across the wound to reform the epithelium, whilst releasing growth factors that stimulate fibroblasts. Fibroblasts in turn migrate and deposit new ECM [54]. Examining BP effects on both cells independently, and together in co-culture, provides distinct evidence of how the soft tissues are affected.

BP treatment has been shown to be toxic to both oral keratinocytes and fibroblasts, and negatively affect cell proliferation and migration, within the concentration range that the oral mucosa is expected to be exposed to [55–57]. A summary of the effects seen on fibroblasts is shown in Table 1, with the effects on keratinocytes shown in Table 2. This review has been limited to *in vitro* studies, as these are able to examine specific cellular mechanisms individually, and does not cover non-BP bone modifying agents (e.g., denosumab) due to the prevalence of BP-related MRONJ, and as limited data exists on the effects of these agents on the oral mucosa.

**Table 1:** A summary of the current literature investigating the 2D effects of pamidronic acid (PA) and zoledronic acid (ZA) on fibroblasts.

**BP**	**Effect**	**Concentration**	**Assays**	**Summary**	**References**
**Viability**					
PA	Decrease	5 μM−25 h 60 μM−48 h 43 μM−72 h 25 μM−96 h 30 μM−168 h	MTT MTS Resazurin	All showed decrease despite differences in concentration and time points	[56, 58–61]
ZA	Decrease	50 μM−24 h 3 μM−48 h 5 μM−72 h 10 μM−96 h 2 μM−168 h 0.16 μM−4 week			[4, 51, 53, 56, 61–68]
	Increase	10 μM−24 h	MTT	Slight positive effect from low concentration	[69]
**Apoptosis**					
PA	Increase	10 μM and above	Annexin V TUNEL Caspase 3	Consistent increase in apoptosis despite differences in concentration	[56, 58, 59, 70]
ZA	Increase	1 μM and above			[4, 60, 64, 66, 68, 71]
	No effect	0.5 μM and above			[70]
**Proliferation**					
PA	Decrease	10 μM and above	^3^[H]thymidine Cell counting Ki67 CFSE	Accuracy of assays and sample size limited	[59, 70]
ZA	Decrease	1 μM and above			[64, 66, 70, 72]
**Migration**					
PA	No effect	30 μM and below	Oris™ stopper assay	Non-toxic, proliferation controlled	[70]
	Decrease	5 μM and above	Scratch Boyden chamber	Toxic concentrations regularly used with no control for proliferation	[56, 60, 67, 71]
ZA	Decrease	30 μM and above			[4, 53, 56, 67]
	No effect	5 μM and below	Transwell Oris™ stopper assay	Non-toxic, proliferation controlled	[70, 72]
**Adhesion**					
ZA	Decrease	30 μM and above	Cytokeratin staining	Limited evidence of reduced adhesion; titanium not representative of MRONJ	[56]
	Decrease on titanium	0.5 μM and above			[73]

*CFSE, carboxyfluorescein succinimidyl ester; MTS, [3-(4,5-dimethylthiazol-2-yl)-5-(3-carboxymethoxyphenyl)-2-(4-sulfophenyl)-2H-tetrazolium], MTT, 3-(4,5-dimethylthiazol-2-yl)-2,5-diphenyltetrazolium bromide; TUNEL, terminal deoxynucleotidyl transferase dUTP nick end labeling*.

**Table 2:** A summary of the current literature investigating the 2D effects of pamidronic acid (PA) and zoledronic acid (ZA) on keratinocytes.

**BP**	**Effect**	**Concentration**	**Assays**	**Summary**	**References**
**Viability**					
PA	Decrease	100 μM−24 h 50 μM−72 h 1 μM−96 h 100 μM−1 week	MTT MTS Resazurin	All showed decrease despite differences in concentration and time points	[52, 55, 58, 74]
ZA	Decrease	5 μM−24 h 3 μM−48 h 1 μM−72 h			[4, 52, 66, 74–77]
	Increase	10 μM−48 h	Resazurin	Slight positive effect from low concentration	[78]
**Apoptosis**					
PA	No effect	100 μM and below	Annexin V TUNEL Caspase 3	Apoptosis in keratinocytes is present in some studies, while others point toward a different pathway	[55, 58, 70]
	Increase	5 μM and above			[52, 74]
ZA	No effect	10 μM			[4]
	Increase	0.25 μM and above			[47, 52, 70, 74, 77]
**Proliferation**					
PA	Decrease	10 μM and above	CFSE	Accuracy of assays and sample size limited	[70]
ZA	Decrease	1 μM and above	Cell counts ELISA		[66, 70, 72, 76]
**Migration**					
PA	No effect	10 μM and below	Oris™ stopper assay	Non-toxic, proliferation controlled	[52, 55, 74]
	Decrease	50 μM and above	Scratch Boyden chamber	Toxic concentrations regularly used with no control for proliferation, results inconsistent	[70]
ZA	Increase	10 μM and below			[4, 78]
	Decrease	100 μM and above			[52, 57, 74]
	No effect	10 μM and below	Transwell Oris™ stopper assay	Non-toxic, proliferation controlled	[70, 72]
**Adhesion**					
ZA	Decrease on titanium	0.5 μM and above	Cytokeratin staining	Limited evidence of reduced adhesion; titanium not representative of MRONJ	[73]

*ELISA, enzyme-linked immunosorbent assay*.

#### Cell Viability

Cell viability provides an approximate measure of the toxicity of a substance and the effects of BPs on oral mucosa cell viability have been extensively studied. Investigations have used fibroblasts and keratinocytes from a variety of sources to assess the effects of BPs over time. Both PA and ZA have been reported to be toxic to fibroblasts and keratinocytes at concentrations clinically relevant to MRONJ patients [4, 51, 52, 55, 56, 58, 59, 64–68, 71, 74–76]. There can often be a lack of clarity as to specific toxic concentrations in literature, with wide concentration ranges between the highest non-toxic and lowest toxic concentrations (e.g., 30 and 100 μM [55]) or few concentrations examined. However, in those papers which have included broader ranges of drugs and half-maximal inhibitory concentrations (IC_50_), there is a strong consensus on the toxicity of PA and ZA on both fibroblasts and keratinocytes.

PA has been demonstrated to be toxic to human oral fibroblasts from 24 h of administration, with lower concentrations causing toxicity after longer the treatment length. For example an IC_50_ of 43 μM was generated at 72 h of treatment in work from our group [61], while at 96 h, Kim et al. demonstrated a 10 μM PA treatment significantly reduced viability [58]. There is some variability within the results, however the toxic effect of PA at physiologically relevant concentrations and over short time scales is clear.

A similar effect has been consistently seen with ZA, and as ZA is a more potent drug, toxicity has been witnessed at lower concentrations. Toxicity has been seen from concentrations as low as 14.7 μM at 48 h with human gingival fibroblasts (HGFs) [62]. Work from our group and Jung et al. demonstrated toxicity from ≈5 μM ZA after 72 h [61, 71]. The toxicity has been studied following 4 weeks of treatment, where concentrations ranging from 0.15625 to 2.5 μM were shown to have a dose dependent, significant effect on viability [51]. As with PA, some inconsistencies exist in the results, with some studies requiring higher concentrations to cause toxicity [60, 68], however the general trend is the same: ZA is toxic to fibroblasts at physiologically relevant concentrations.

Keratinocyte viability in the presence of both drugs has also been examined. More inconsistency exists in the keratinocyte studies than with fibroblasts, as a wider range of cell sources have been tested, including cells of both primary and immortalized nature, from oral and skin sources, and human and murine origin [52, 55, 78, 79]. Despite this the trend of PA and ZA toxicity persists. Keratinocyte toxicity is generally reported at slightly higher concentrations than fibroblast toxicity, and ZA is approximately twice as toxic as PA [74, 79]. Again, over time, lower concentrations are required to cause toxicity, for example 50 μM ZA at 24 h and 5 μM ZA at 72 h in two studies from Pabst et al. [52, 74]. This demonstrates that persistent exposure to BPs has the potential to cause damage to the oral epithelium as BPs are released into the oral environment in MRONJ, and offers an explanation for why necrotic bone exposure persists.

Conversely, literature has indicated a positive effect on cell viability caused by subtoxic nanomolar concentrations of ZA over short time scales, with the effect seen in both keratinocytes and fibroblasts [63, 69, 78] (an effect commonly observed in *in vitro* experiments of toxic substances at very low concentrations). In keratinocytes, Renò et al. suggested this may be due to a downstream effect of mevalonate pathway inhibition [78]. In fibroblasts the effect was unexplained, though Manzano-Moreno et al. also noted a reduced expression of proteins, that would ultimately reduce the oral mucosa wound healing capability longer term [69]. Any potential benefit, therefore would be likely to not be found in MRONJ patients where exposure is longer term and at higher concentrations.

Arai et al. also treated keratinocytes with ZA at nanomolar concentrations, noting no beneficial effect [75]. They did, however, indicate that local calcium levels could drastically affect the potency of BPs. They theorized that due to the BP binding affinity for calcium, increasing cellular calcium levels would thereby increase the amount of BP acting upon the cell. This is an important consideration, and has potential to influence the BP effect on the oral epithelium, which naturally contains a concentration gradient through the keratinocyte layers with the superficial layers having the highest calcium concentration [80].

Toxicity is an obvious mechanism by which BPs cause persistent exposure to necrotic bone in MRONJ, and the data clearly demonstrates that even a medium potency BP such as PA reduces cell viability in a significant manner, when tested with clinically relevant concentrations and short time periods. ZA is toxic toward the lower estimates of the expected mucosal exposure concentration, and in some studies within the salivary concentration defined by Scheper et al. [47], confirming what is known regarding MRONJ incidence with low vs. high potency BPs.

#### Apoptosis

As well as confirming that BPs reduce viability to cells of the oral mucosa, the mechanism by which BPs cause cell death has been investigated. Apoptosis is the programmed death of a cell, and can occur in response to certain external triggers, with the inhibition of the mevalonate pathway known to cause apoptosis [81].

BPs have been demonstrated to increase fibroblast apoptosis, however, as with viability, some inconsistency regarding the specific concentrations to cause this effect exists. PA has been shown to increase fibroblast apoptosis in a dose dependent manner after 72 h of treatment from concentrations as low as 0.1 μM [59]. With ZA, apoptosis has been reported to be triggered from 48 h onwards, with significant apoptosis recorded with concentrations as low as 30 μM [64, 68, 71]. Contrarily, while Cozin et al. did observe toxicity in HGFs with both PA and ZA, they indicated only PA led to increased caspase activity [56], which correlates to work from our group which indicated higher apoptosis levels seen following toxic PA treatment compared to ZA treatment [70].

While fibroblast apoptosis is a fairly consistent effect throughout the literature, there is less clarity regarding keratinocytes. This may be in part again due to the wider range of sources of tested cells. There is also data to suggest the mechanism by which BPs cause cell death to keratinocytes is different to fibroblasts [4, 55, 58].

**Figure 2:**
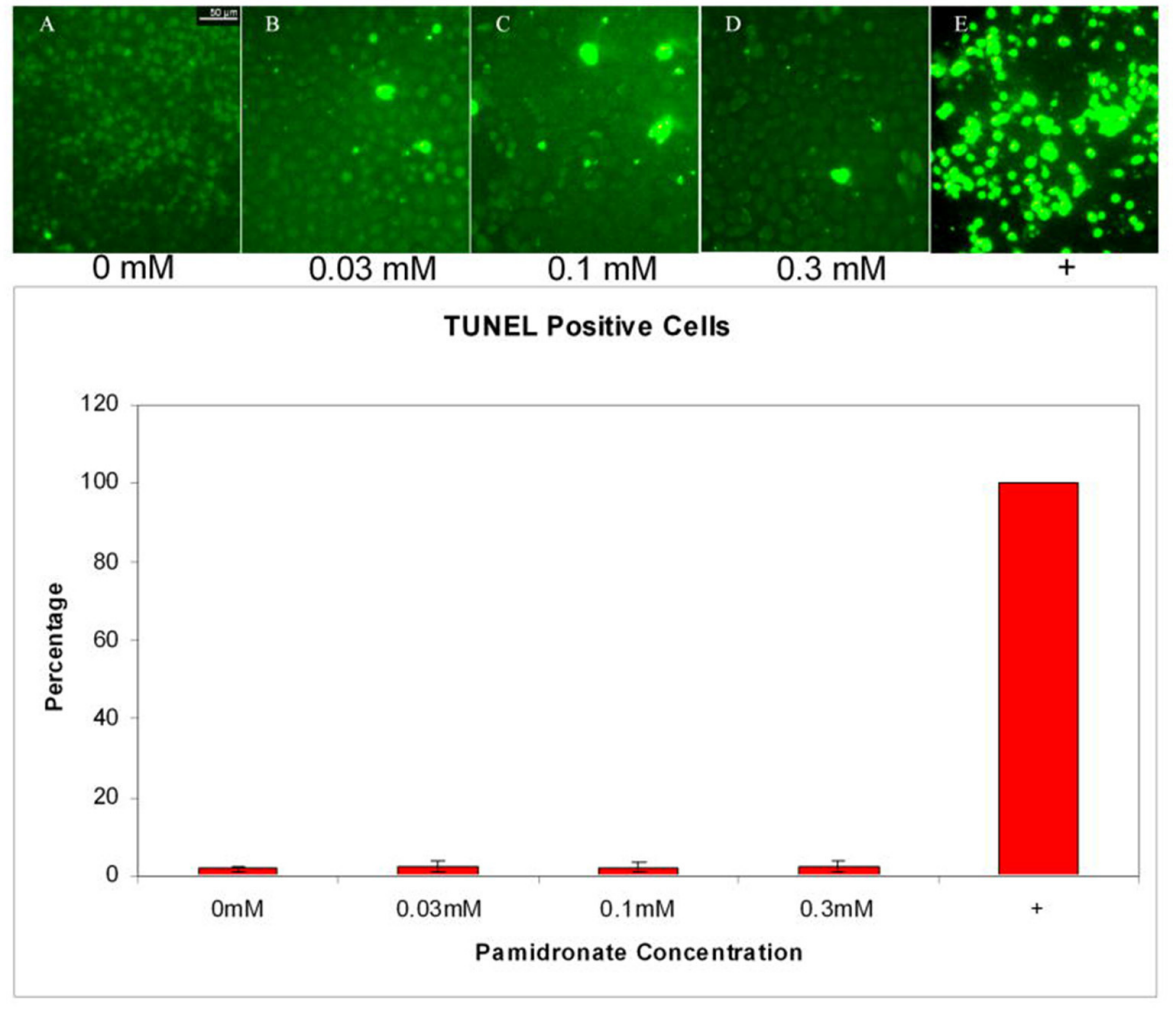
Apoptosis in oral keratinocytes incubated with pamidronate. TUNEL assay performed on cells incubated with 0.03, 0.1, and 0.3 mM pamidronate did not increase the percentage of TUNEL positive cells when compared to non-treated cells. Staurosporine and cycloheximide treated positive control cells were all apoptotic by 24 h. Reproduced with permission from Landesberg et al. [55].

Whilst some studies have highlighted a significant increase in keratinocyte apoptosis through treatment over similar time scales and PA and ZA concentrations to the viability studies [52, 57, 66, 70, 74, 77], there is no consensus over the effect. The two studies by Pabst et al. disagreed as to whether PA or ZA led to higher apoptosis levels [52, 74] while Scheper et al. indicated that apoptosis occurred at a higher rate in keratinocytes than in fibroblasts [66]. Other papers disagree with this entirely. Both Ravosa et al. and Kim et al., who compared apoptosis levels in fibroblasts and keratinocytes when treated with ZA and PA, respectively, found no significant keratinocyte apoptosis [4, 58], and Landesberg et al. did not see significant keratinocyte apoptosis after PA treatment, shown in Figure 2 [55]. Kim et al. suggested that BPs led to the cell death of keratinocytes by triggering early senescence, rather than apoptosis as in fibroblasts, and successfully stained for markers of senescence to confirm their theory [58]. Further work to fully elucidate the cytotoxic mechanisms of PA and ZA on keratinocytes could give a clearer picture of how MRONJ develops and progresses, and therefore have a high impact in the field.

#### Proliferation

Cell proliferation is a key part of wound healing and restoration of the mucosal barrier. It has been suggested that another potential mechanism by which BPs prevent oral mucosa wound healing is through decreasing proliferation. The mevalonate pathway has been linked to cellular proliferation [82] and therefore this presents an interesting area of study.

Several papers in this literature review purported to have reported on effects on proliferation, using MTT or similar metabolic activity assays [4, 51, 55, 56, 69, 78]. However, as these assays in fact measure metabolic activity, not proliferation, those results have been discussed as cell viability previously in this article. The review of literature in this section only relates to those papers that have directly measured proliferation.

PA and ZA have both been shown to reduce proliferation of human oral fibroblasts at clinically relevant concentrations. Soydan et al. found PA reduced proliferation in a dose dependent manner with concentrations ranging from 0.1 to 100 μM [59]. Agis et al. noted a similar effect with fibroblasts treated with ZA, with 30 and 100 μM concentrations significantly reducing proliferation [64], while ZA concentrations as low as 1 and 3 μM were found to reduce proliferation by Scheper et al. [66].

Keratinocyte proliferation has also been found to be affected by BPs, in a wide range of studies consisting of different methodologies. Our group has indicated that 10 μM PA and above significantly reduced keratinocyte proliferation [70]. ZA has been shown to significantly reduce proliferation from 48 h, with subtoxic concentrations having this effect [66, 70, 72]. Work from Ohnuki et al. examined the keratinocyte cell cycle, and indicated the effect on proliferation was due to the cell cycle being arrested in the S phase by damaging DNA and thereby reducing the expression of proteins involved in cell cycle regulation [76]. This effect was also seen in work from Kim et al. with both keratinocytes and fibroblasts treated with PA [58]. Their data again indicated the arrest of keratinocytes in the S phase of the cell cycle, where fibroblasts treated with 50 μM PA were largely in the sub-G1 phase. This suggests that keratinocytes were prevented from completing their cell cycles while fibroblasts became apoptotic when treated with BPs, again highlighting nuance in the response from the oral mucosa to BP exposure.

There is clear evidence that PA and ZA affect the proliferation of oral fibroblasts and keratinocytes, which indicates one mechanism by which BPs prevent the full healing of the oral mucosa and contribute to the prolonged exposure of necrotic bone in MRONJ patients.

#### Migration

In MRONJ, the necrotic bone is exposed through non-healing lesions in the oral mucosa. As migration is a key part of oral mucosa wound healing, the effects of BPs on cellular migration have been examined. Migration is often studied *in vitro* with a scratch assay, whereby cells are cultured to confluence, before a sterile pipette tip is scratched across the cell monolayer to create a gap between the cells [83]. Migration across this “wound” is then assessed over time.

The effects of BPs on fibroblast migration have been investigated in several studies. Clinically relevant concentrations of both PA and ZA have been indicated to reduce migration, with ZA again more potent than PA [4, 53, 56, 60, 67, 71, 72]. Less of a consensus exists in literature with keratinocytes. PA and ZA have been demonstrated to slow migration, however the variety of cell sources means there is a wide variation between the reported concentration of this effect, ranging from 100 nM to 50 μM [52, 57, 74, 77]. ZA has conversely been shown to increase keratinocyte migration, in studies by Renò et al. and Ravosa et al. with concentrations up to 10 μM ZA [4, 78]. Alternatively, Landesberg et al. reported PA concentrations from 3 to 100 μM had no effect on murine oral keratinocyte migration over 96 h [55]. Only when cells were pre-treated with 100 μM for 72 h prior to the experiment was any effect seen, and this concentration was reported to be toxic at this exposure time in the same article [55].

Whilst these results suggest effect of BPs on cell migration there are limitations in the experimental design which reduce the reliability of this data. The reporting of toxicity and reduced migration from the same concentrations at the same time points is common [52, 53, 56, 60, 67, 71, 74, 77], likely pointing to lack of wound closure through cell toxicity, rather than a separate migration effect. While images of the cells are often not included, Cozin et al., Paulo et al., Wang et al. and Yuan et al. do include images and some discrepancies appear. For example Cozin et al. show cells that do not appear to be alive, with either side of the wound no longer well-formed [56], particularly when treated with ZA. Wang et al. demonstrate well-formed edges of cells which appear healthy after 72 h of ZA treatment, despite defining their chosen concentrations as toxic earlier in their paper [77]. These inconsistencies are not explained.

As previously described, proliferation is a key part of wound healing, and therefore, to truly assess migration effects, proliferation must be prevented in the experimental set up which is not included in any of the above articles.

The migration of immortalized human foreskin fibroblasts and immortalized oral keratinocytes (OKF6/TERT-2) cells was studied in the presence of sub-toxic levels of ZA by McLeod et al. [72]. Using sub-toxic levels of the drug ensures that toxicity will not prevent the wound from closing, and an assay which measures migration alone, no effect on migration was seen over 24 h. In work from our group, cells were pre-treated with mitomycin C to prevent cell proliferation, before a migration assay was performed with sub-toxic BP concentrations [70]. PA and ZA caused no effect on the migration of oral fibroblasts or keratinocytes. This suggests that while BPs do cause toxicity and prevent cellular proliferation, they do not have a direct effect on cell migration.

#### Adhesion

Bisphosphonates have been shown to affect the adhesion of a wide variety of cell types including smooth muscle cells [84], endothelial cells [85–87] and cancer cells [88]. This is hypothesized to be due to the BP effect on the mevalonate pathway, preventing proteins from acting at the correct location in the cell [30]. In adhesion, cytoskeletal organization is prevented through an effect on the signaling pathway including Focal Adhesion Kinase (FAK) [89]. The phosphorylation of FAK is critical for the assembly and disassembly of focal adhesions [90].

**Figure 3:**
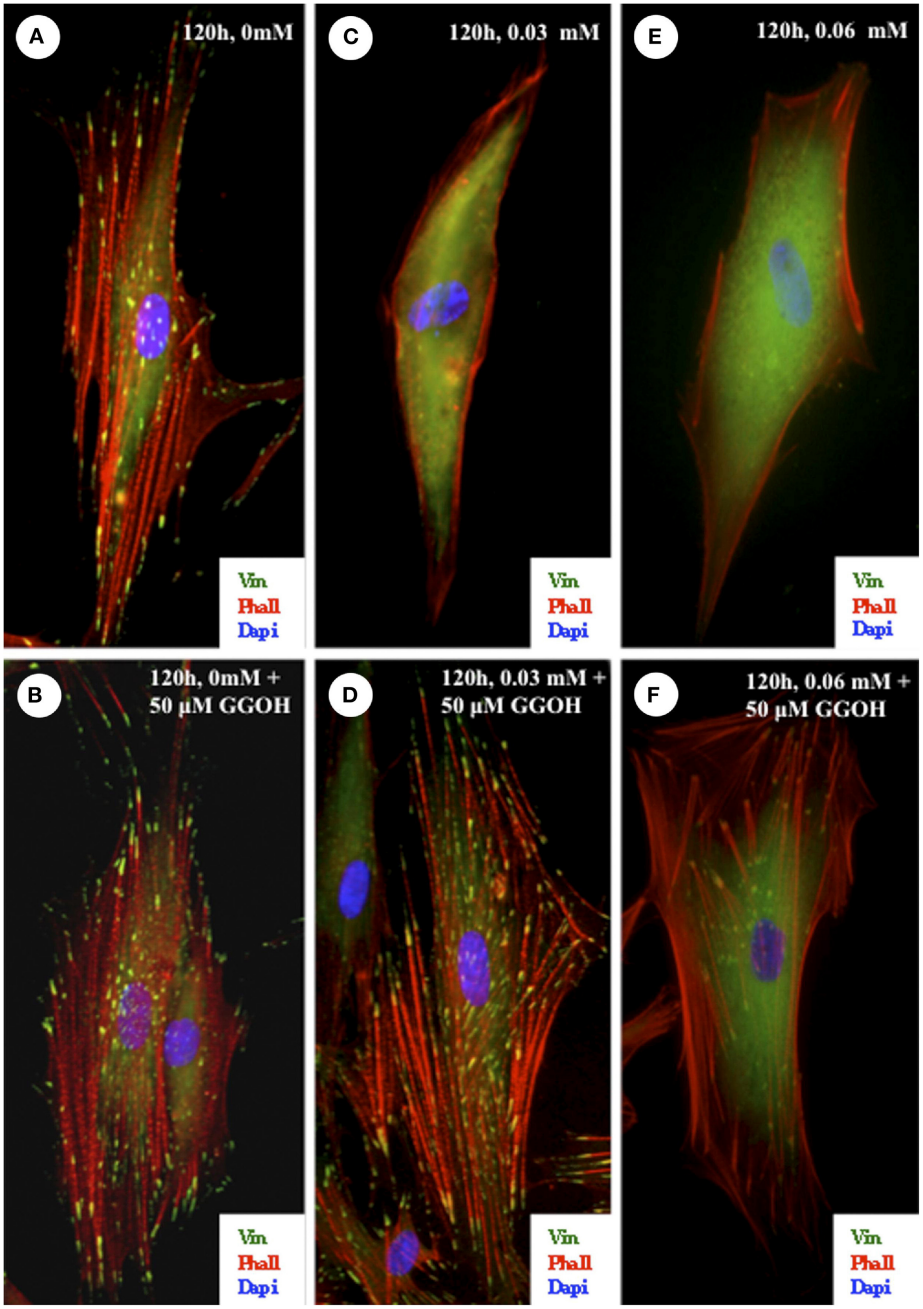
Effect of zoledronate on integrin-mediated cell-substratum adhesions of oral fibroblasts. Cells were plated on fibronectin-coated coverslips and exposed to 0.03- or 0.06-mmol/L zoledronate with or without the addition of 50-μmol/L GGOH. The cells were fixed after 120 h and then stained with vinculin and phalloidin to visualize the focal adhesions and actin cytoskeleton. **(A,B)** In cells that were untreated or treated with 50-μmol/L GGOH alone, there was a robust actin cytoskeletal network associated with numerous focal adhesions. **(C,E)** In cells that were exposed to 0.03- or 0.06-mmol/L zoledronate, there were very few F-actin bundles and a corresponding loss of focal adhesions. **(D)** The addition of 50-μmol/L GGOH to cells treated with 0.03-mmol/L zoledronate was able to completely rescue both the focal adhesions and the actin stress fibers. In cells treated with 0.06-mmol/L zoledronate and 50-μmol/L GGOH, the focal adhesions and actin cytoskeleton network were also restored **(F)** but not to the same extent as shown in **(D)**. (Original magnification, ×100). Reprinted with permission from Cozin et al. [56].

ZA has been demonstrated to decrease FAK phosphorylation in several cell types *in vitro* [84, 85, 88, 91], in conjunction with a reduction in adhesion, which further strengthens the theory of a link between BPs and reduced cellular adhesion. Cozin et al. demonstrated that 30 μM concentrations of ZA lowered fibroblast adhesion alongside its toxic effect, shown in Figure 3 [56]. Cells were stained to visualize focal adhesions and F-actin bundles in the cytoskeleton, and these were seen to be reduced when cells were treated with the BP.

ZA has also been shown to prevent some integrin-mediated adhesion, specifically α_v_β_3_ [87, 88], α_v_β_5_ [87] and α_v_β_6_ [92]. However, these integrins are not strongly expressed in the epithelium of healthy oral mucosa, with α_v_β_5_ expressed weakly and α_v_β_6_ only expressed in response to wounding [93]. As MRONJ develops following wounding of the epithelium, this α_v_β_6_ pathway could be involved in BP related changes in oral epithelial cell adhesion, but has yet to be explored. Alendronic acid has also been linked to a reduction in epithelial adhesion, with biopsies from long term oral BP patients showing changes in desmoglein-1, a protein involved in the adhesion of keratinocytes in the oral epithelium [94]. The oral epithelium from those taking alendronate was still intact however the superficial layers of the epithelium expressed less desmoglein-1 compared to samples from individuals not exposed to BPs. This suggests that even in patients who do not display MRONJ symptoms, the epithelium may already be weaker and more susceptible to lesions forming, which may play a role in the development and progression of MRONJ.

The effects of ZA on gingival fibroblast and HaCaT adhesion to titanium were tested by Basso et al. [73]. In their study 0.5 and 1 μM treatments of ZA led to fewer cell numbers and less actin and homing cell adhesion molecule staining over 48 h. However, as this was investigating their effects on titanium it is less relevant to MRONJ patients.

Due to what is known about the BP mechanism of action, and the role in adhesion noted in other cell types, this offers an interesting area and further study is required to determine whether BPs affect the adhesion of cells in the oral mucosa, and whether this plays a role in MRONJ.

#### 3D *in vitro* Effects of BPs

To gain a greater understanding of how BPs affect oral tissues, a variety of 3D oral mucosa models have been studied in the presence of BPs. These models contain both oral fibroblasts and keratinocytes together on a scaffold in a system analogous to *in vivo* tissue which allow for more physiologically relevant data to be obtained. Cells are known to behave differently in 3D culture systems as cross-talk and signaling between different cell types is critical to many biological processes including proliferation, migration and wound healing [95]. 3D models can also be used to investigate different aspects of epithelial development, integrity and repair.

The effects of BPs have been studied at different phases of tissue growth and regeneration, examining developing and healing tissues, alongside healthy tissue. This allows for examination of both the BP contribution to prolonged bone exposure through the prevention of healing, alongside any potential BP effect on the healthy tissue of at-risk patients.

McLeod et al. used oral mucosa models to study the effects of sub-toxic BP levels on epithelial development [72]. Oral mucosa models are seeded with one epithelial layer which then stratifies over time. In their study, models were treated with 1 μM ZA for 7 days in culture during the stratification process, and no differences were seen. With or without BP treatment, the epithelium was able to stratify as normal. Our group has demonstrated concentrations above 1 μM ZA and 50 μM PA reduced the epithelial thickness and metabolic activity of similar models [70].

**Figure 4:**
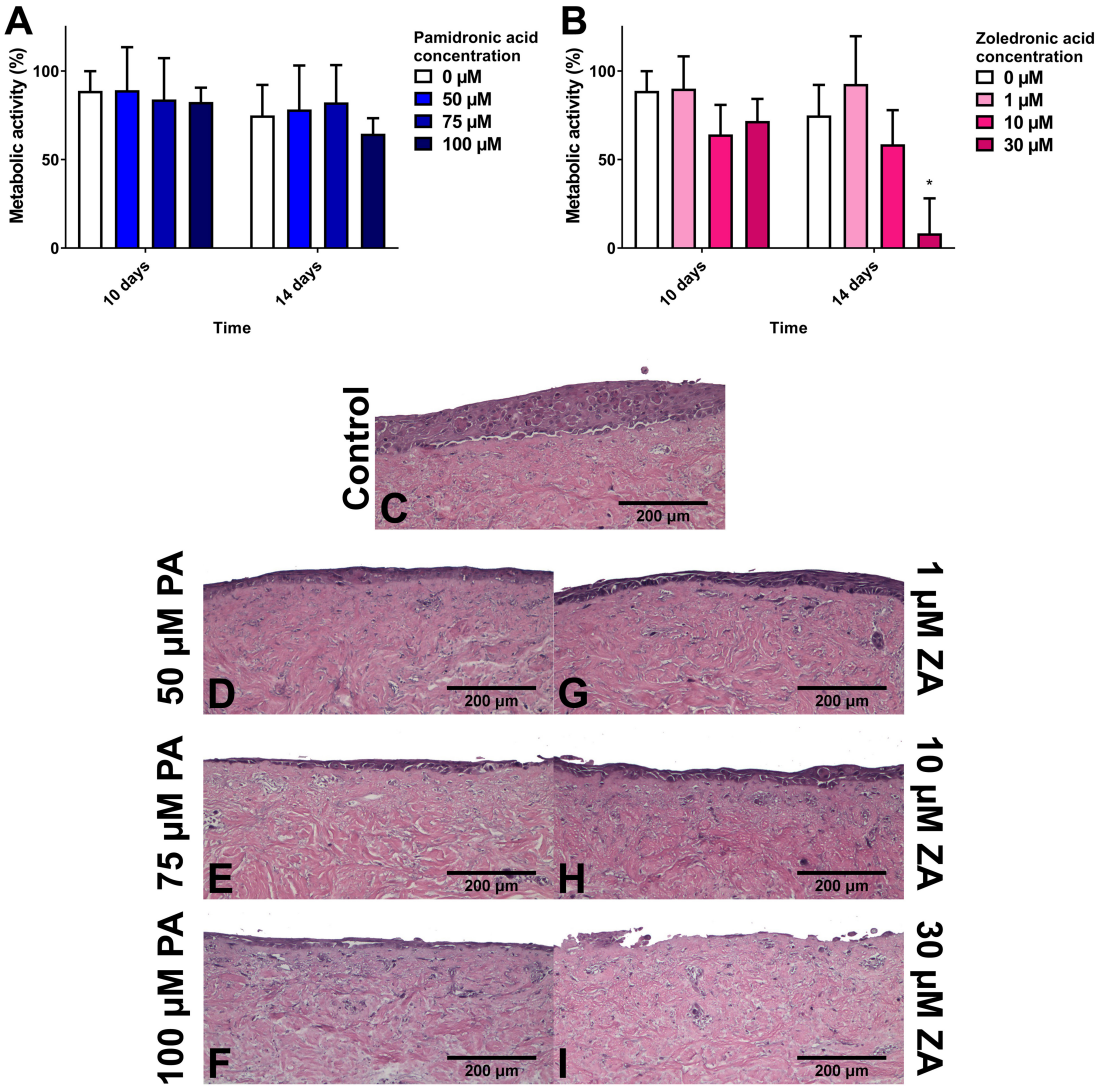
The metabolic activity of oral mucosa models when treated with **(A)** pamidronic acid and **(B)** zoledronic acid for 7 days after prior culture at air liquid interface (ALI) in control medium for 7 days, measured with a resazurin assay. A blank well reading was subtracted before values were normalized to day 7 value for each model, defining day 7 values as 100% (not shown). *N* = 3, *n* = 3. Error bars = SD. Statistical significance against 0 μM at each time point indicated by *p ≤ 0.05. **(C–I)** H & E-stained sections of oral mucosa models seeded with human oral fibroblasts and immortalized human oral keratinocytes cultured at ALI for 7 days in control medium, then treated with **(C)** control medium; **(D)** 50 μM, **(E)** 75 μM, and **(F)** 50 μM pamidronic acid; **(G)** 1 μM, **(H)** 10 μM, and **(I)** 30 μM zoledronic acid, respectively, for 7 days. Representative images used. Reproduced from Bullock et al. [61] under a CC BY license.

To examine healthy mucosa, models were cultured in standard media and epithelia allowed to stratify before BP treatment. PA treatment has been demonstrated to reduce epithelial thickness in studies from our group, and Kim et al. [58, 61]. Kim et al. witnessed a thinner basal layer and intact keratinised layers, and suggested that BP treatment may speed up keratinocyte differentiation. Our work, shown in Figure 4, examined the metabolic activity of these models, which interestingly showed no difference between any BP treatment or non-treated controls despite changes in epithelial thickness. ZA was also examined in our study, and a much more pronounced effect was seen, with 10 μM ZA thinning the epithelia and 30 μM treatment almost entirely removing the epithelium [61]. Metabolic activity was significantly reduced following 30 μM treatment in models using OKF6/TERT-2 cells, and both 10 μM and 30 μM in models with primary keratinocytes [70].

Other studies have further examined ZA treated models to identify the mechanisms of the epithelial effects with contrasting results. A reduction in epithelial thickness following ZA treatment was seen by Ohnuki et al. when keratinocytes alone were seeded in a 3D model, and allowed to stratify before treatment [76]. As mentioned previously in this article, further testing showed basal cells proliferated less with BP treatment, due to the drug arresting cells in the S phase of the cell cycle. Conversely, Bae et al. found the superficial layers were most affected [96]. They treated 3D models, which also included a rat calvariae bone layer, with either a less potent, fluorescently labeled version of ZA, or standard ZA, to examine drug localization. The standard, toxic version of ZA removed the superficial layers of the epithelium, whilst imaging showed the fluorescently labeled ZA was located in the superficial layers of the epithelium, even though the mucosal section of the model was never in direct contact with the drug-containing media. The calcium content of the superficial epithelial layers is higher than the basal layers [80], which therefore suggests that even within the epithelium, the calcium binding affinity of BPs plays a role in its effects. It was theorized that low levels of BPs may localize to the soft tissue prior to MRONJ being triggered and further BPs could be released following soft tissue damage. Clear evidence of these mechanisms could allow for more targeted treatments for MRONJ and this offers an obvious area for further study.

The effects of ZA on wound healing in 3D have been examined. Saito et al. seeded models and allowed them to stratify, before creating a wound using scalpel blades [92]. ZA slowed the healing significantly, which they concluded was due to a reduction in proliferation, and reduction in expression of the αvβ6 integrin, indicating BPs prevent the intracellular signaling mechanisms which promote wound healing. Kim et al. cultured models before wounding with a biopsy punch on culture day 7 [58]. PA prevented these wounds from healing and further examination again pointed to a lack of proliferation. These studies represent a promising start in examining the effects of BPs on healthy mucosa, but more depth is required to fully elucidate the BP effect on oral mucosa re-epithelialisation in MRONJ patients.

In conclusion, whilst BPs are excellent treatments for osteoporosis and cancers which have metastasised to bone, MRONJ as a side effect cannot be ignored. MRONJ has a significant effect on the quality of life of patients and is a growing problem as prescriptions for high potency BPs increase. Existing treatment methods have shown limited success with many patients relying on long term symptom management rather than disease resolution. Bisphosphonates are responsible for the majority of MRONJ cases and, as shown in this review, have far reaching effects on many different cellular processes linked to wound healing and tissue repair.

The studies reviewed here which investigated the *in vitro* effects of BPs on fibroblasts and keratinocytes, demonstrate clear negative effects on cell viability, apoptosis and proliferation. These effects occur at BP concentrations thought to be clinically relevant based on estimations and measurements on salivary and bone BP concentrations [46, 47]. The evidence for an effect on migration is not as convincing and we hypothesize the impairment of re-epithelialisation is due to a reduction in cell proliferation and cytotoxicity, rather than cell migration. The effects of BPs on adhesion are less clear however there is compelling evidence for further study due to the reported effect on the mevalonate pathway and studies described above. In 3D oral mucosa models, BPs reduce the thickness of new and established epithelia, and prevent wound healing. These mechanisms are likely to make the oral mucosa more susceptible to damage and contribute to the progression of MRONJ. As a result MRONJ remains a difficult clinical challenge to overcome. Ultimately it is hoped the increased understanding of the underlying cellular mechanisms, as presented in this study, will lead to new treatment targets and improved outcomes for MRONJ patients in the future.

## Author Contributions

VH, CM, and AM: conception and design. GB: data acquisition, analysis, and drafting of manuscript. GB, VH, CM, and AM: critical revision of manuscript and final approval of manuscript. All authors contributed to the article and approved the submitted version.

## Funding

This research was funded by the Engineering and Physical Sciences Research Council (EPSRC) through a DTA studentship awarded to the University of Sheffield, an EPSRC Doctoral Prize Fellowship awarded to Dr. George Bullock (Grant code: X/013296), and an EPSRC Impact Acceleration Account (Grant code: X/167000).

## Conflict of Interest

The authors declare that the research was conducted in the absence of any commercial or financial relationships that could be construed as a potential conflict of interest.

## Publisher's Note

All claims expressed in this article are solely those of the authors and do not necessarily represent those of their affiliated organizations, or those of the publisher, the editors and the reviewers. Any product that may be evaluated in this article, or claim that may be made by its manufacturer, is not guaranteed or endorsed by the publisher.
